# Siglec15 is a prognostic indicator and a potential tumor-related macrophage regulator that is involved in the suppressive immunomicroenvironment in gliomas

**DOI:** 10.3389/fimmu.2023.1065062

**Published:** 2023-05-30

**Authors:** Jinchao Wang, Linzong Xu, Qian Ding, Xiaoru Li, Kai Wang, Shangchen Xu, Bin Liu

**Affiliations:** ^1^ Department of Neurosurgery, Shandong Provincial Hospital Affiliated to Shandong First Medical University, Shandong First Medical University, Jinan, China; ^2^ Graduate School of Medicine, Shandong First Medical University, Jinan, China; ^3^ Tumor Research and Therapy Center, Shandong Provincial Hospital, Shandong University, Jinan, China; ^4^ Department of Gastroenterology, Shandong Provincial Hospital Affiliated to Shandong First Medical University, Shandong First Medical University, Jinan, China; ^5^ Department of Critical Care Medicine, Shandong Provincial Hospital Affiliated to Shandong First Medical University, Shandong First Medical University, Jinan, China

**Keywords:** SIGLEC15, gliomas, immune checkpoint, prognostic indicator, macrophages

## Abstract

**Background:**

Siglec15 is rising as a promising immunotherapeutic target in bladder, breast, gastric, and pancreatic cancers. The aim of the present study is to explore the prognostic value and immunotherapeutic possibilities of Siglec15 in gliomas using bioinformatics and clinicopathological methods.

**Methods:**

The bioinformatics approach was used to examine Siglec15 mRNA expression in gliomas based on TCGA, CGGA, and GEO datasets. Then, the predictive value of Siglec15 expression on progression-free survival time (PFST) and overall survival time (OST) in glioma patients was comprehensively described.The TCGA database was screened for differentially expressed genes (DEGs) between the high and low Siglec15 expression groups, and enrichment analysis of the DEGs was performed. The Siglec15 protein expression and its prognostic impact in 92 glioma samples were explored using immunohistochemistry Next, the relationships between Siglec15 expression and infiltrating immune cells, immune regulators and multiple immune checkpoints were analysed.

**Results:**

Bioinformatics analyses showed that high Siglec15 levels predicted poor clinical prognosis and adverse recurrence time in glioma patients. In the immunohistochemical study serving as a validation set, Siglec15 protein overexpression was found in 33.3% (10/30) of WHO grade II, 56% (14/25) of WHO grade III, and 70.3% (26/37) of WHO grade IV gliomas respectively. Siglec15 protein overexpression was also found to be an independent prognostic indicator detrimental to the PFST and OST of glioma patients. Enrichment analysis showed that the DEGs were mainly involved in pathways associated with immune function, including leukocyte transendothelial migration, focal adhesion, ECM receptor interaction, and T-cell receptor signaling pathways. In addition, high Siglec15 expression was related to M2 tumor-associated macrophages (TAMs), N2 tumor-infiltrating neutrophils, suppressive tumor immune microenvironment, and multiple immune checkpoint molecules. Immunofluorescence analysis confirmed the colocalization of Siglec15 and CD163 on TAMs.

**Conclusion:**

Siglec15 overexpression is common in gliomas and predicts an adverse recurrence time and overall survival time. Siglec15 is a potential target for immunotherapy and a potential TAMs regulator that is involved in the suppressed immunomicroenvironment in gliomas.

## Introduction

Glioma is the most prevalent primary central nervous system (CNS) malignancy, accounting for about 80% of all primary malignant brain tumors (1). Despite the variety of treatments available for glioma in modern medicine, it remains a fatal tumor disease with a very poor prognosis. Therefore, identifying new and effective treatment strategies is an urgent task for glioma treatment.

Immunotherapy is emerging as a novel treatment modality and improvs the prognosis of various types of cancers, including CNS malignancies (2, 3). Important advancement in cancer immunotherapy includes immune stimulation, adoptive T-cell transfers, vaccination strategies, and checkpoint inhibitors (4). Nevertheless, checkpoint inhibitors, such as anti-CTLA4, anti-PD-1 and anti-PD-L1, have poor clinical efficacy in glioma (4). Therefore, further exploration of glioma-related immunotherapeutic targets to improve the effectiveness of immunotherapy are urgently required.

The sialic acid-binding immunoglobulin-like lectin (Siglec) family is the largest family of vertebrate lectins known to recognize sialylated glycans. Siglec15 is a type I transmembrane protein containing only a V-set immunoglobulin (Ig) structural domain and a C2-set immunoglobulin that is highly similar to PD-L1 (5), in contrast to other members of the Siglec family (6). Siglec15 plays an important role in maintaining immune homeostasis, and its dysregulation may lead to cancer progression by suppressing T cells through different pathways (5, 7). Siglec-15 is up-regulated in bladder, colon, endometrial, kidney, lung and thyroid cancers and may have prognostic significance (8). In a phase I clinical trial for patients with advanced non-small cell lung cancer (NSCLC), improved outcomes for patients treated with a Siglec-15 inhibitor (NC318) (NCT03665285) were observed (NCT03665285). However, the role of Siglec15 in glioma remains unreported, and a better understanding of the function of Siglec15 in glioma will contribute to the further development of cancer immunotherapies.

In the present study, a comprehensive analysis of Siglec15 mRNA and protein levels in gliomas is presented herein by bioinformatics methods and immunohistochemistry, respectively. In addition, prognostic significance of the Siglec15 mRNA and protein overexpressions in glioma patients were analysed. Finally, the potential mechanism of Siglec15 in the regulation of the immunosuppressive microenvironment of gliomas was explored using bioinformatics methods. These findings imply that Siglec15 is a novel prognostic marker and a potential target for anti-tumor associated macrophages (TAM) immunotherapy in gliomas.

## Methods

### Public data collection and analysis

The GTEx database was used to retrieve the RNA-seq data for normal brain tissues (9). A search of the Cancer Genome Atlas (TCGA) database for fragments per kilobase million (FPKM) values of RNA-seq data and the respective clinical information yielded 698 samples. A total of 693 samples with FPKM values of the RNA-seq data and their clinical background was obtained from the Chinese Glioma Genome Atlas (CGGA) database. The inclusion criteria were as follows: 1) the average of the repeated sequencing downloaded from the database were calculated; 2) samples selected for analysis must contain complete clinical information about the patient, including survival time, survival status, age, and gender. Samples without any of these clinical information were excluded. The final number of glioma samples downloaded from the TCGA database was 703, of which 670 met our criteria. 693 cases were downloaded from the CGGA database, all of which met our criteria. Data which only contain mRNA expression of glioma tissues and normal brain tissues obtained from the NCBI Gene Expression Omnibus (GEO, GSE50161, and GPL570 platform) were then applied for supplementary validation.

### Siglec15 expression analysis

Data from the TCGA and CGGA databases were used to explore the relationship of Siglec15 transcript expression with age, WHO grade, isocitrate dehydrogenase (IDH) status, 1p/19q codeletion and primary treatment outcome. Statistical analysis was conducted using R software (version 4.1.3/3.6.3) and visualisation was carried out using the “ggplot2” package.

### Survival analysis

Kaplan−Meier survival analysis was performed to determine the correlation between Siglec15 expression levels and progression-free survival (PFS) or overall survival (OS) in glioma patients. The glioma cohort was divided into two groups by median Siglec15 mRNA expression (high expression group: 50%–100%; low expression group: 0%–50%). Moreover, to perform subgroup analyses on OS, glioma patients were grouped according to their clinical characteristics. The “survival” and “survminer” packages were used for statistical analysis and visualization, respectively.

### Patient selection

In the validation study, 95 consecutive patients with pathologically confirmed gliomas at Shandong Provincial Hospital Affiliated to Shandong First Medical University between February 2008 and August 2019 were included. Preoperative Karnofsky performance score of each patient was >70. We selected patients who had not received chemotherapy or radiotherapy prior to surgery and for whom preoperative and postoperative CT and/or MRI could be retrieved. Pathological sections of the selected patients were re-evaluated and reclassified by two pathologists according to the criteria of the new World Health Organization classification (2021) (10). The demographic data and tumour characteristics of all samples are shown in **Table 1**. We understand from the pathology department that the hospital only uses immunohistochemistry to routinely examine IDH1/2, while the examination of some other molecular indicators is at the discretion of the clinician in consultation with the patient. This study was examined and approved by the Research Ethics Committee of Shandong Provincial Hospital Affiliated to Shandong First Medical University. The normal brain tissue in this study were from 6 patients with spontaneous cerebral haemorrhage. The study was conducted after written consent was obtained from all patients or their legal representatives.

**Table 1:** Characteristics of patients with glioma from TCGA and CGGA database.

	TCGA	CGGA
Number of patients (n, %)	670 (100)	693 (100)
Age (y), median (range)	46 (14-89)	43(11-76)
Gender (n, %)		
Male	386 (57.61)	398 (57.43)
Female	284 (42.39)	295 (42.57)
Histological grade (n, %)		
WHO II	216 (32)	188 (27.13)
WHO III	237 (35)	255 (36.80)
WHO IV	160 (24)	249 (35.93)
N/A	57 (9)	1 (0.14)
IDH status (n, %)		
Mutation	424 (63.28)	356 (51.37)
Wildtype	478 (68.98)	286 (41.27)
N/A	70 (10.10)	51 (7.36)
1p/19q (n, %)		
Co-deletion	168 (25.07)	145 (20.92)
Non-codeletion	496 (74.03)	478 (68.98)
N/A	6 (0.90)	70 (10.10)

### Immunohistochemistry and immunohistochemical assessment

The Envision PV-style two-step method (PV-9000 Polymer Detection system, Zhongshan Goldenbridge Biotechnology, Beijing, China) was used for immunohistochemical assessment. Formalin-fixed paraffin-embedded tissue sections (4 µM thick) were baked, dewaxed and rehydrated. Heat-induced antigen recovery was performed [EDTA antigen repair solution (pH 9.0) was maintained in a water bath at 98°C for 20 minutes] and endogenous peroxidase activity was then quenched. Primary antibody (anti-Siglec15, ab198684, Abcam, 1:35) was applied overnight at 4°C and then rewarmed at 37°C for 30 min, followed by rewarming at 37°C for 25 min using Polymer Helper (Zhongshan Goldenbridge Biotechnology), followed by 25min at 37°C using polyperoxidase anti-goat IgG (Zhongshan Goldenbridge Biotechnology). Diaminobenzidine and hematoxylin were used as a substrate for specific antibody localization and a nuclear stain, respectively. Siglec15 sections were scored and examined by two independent observers without knowledge of the clinicopathological background of the patient’s samples. When different evaluations were identified, sections were reevaluated simultaneously by observers using a double-headed microscope. Human medulloblastoma served as a positive control. Slides were incubated with phosphate buffered saline in place of the primary antibodies to serve as a negative control.

A total immunostaining score (TIS) was calculated by dividing a proportion score (PS) by an intensity score (IS) to assess Siglec15’s immunoreactivity (11). The PS represents the estimated percentage of positively stained cells (0, none; 1, <10%; 2, 10–30%; 3, 31–50%; 4, >50%). The IS stands for the estimated staining intensity in comparison with control cells (0, no staining; 1, weak; 2, moderate; 3, strong). The TIS (TIS = PS × IS) ranges from 1 to 12 with only nine possible values (0, 1, 2, 3, 4, 6, 8, 9, and 12). Expression of Siglec15 was analysed as a dichotomous covariate in survival analysis, with high Siglec15 (TIS > 4) versus low Siglec15 expression (TIS ≤ 4) (11). In each sample, 500 tumour cells were counted in six independently stained areas with evenly distributed immunopositive staining.

### Operating characteristic curve (ROC) analysis of Siglec15

Time-dependent subject ROC analysis was carried out with the use of the “timeROC” package to illustrate the validity of Siglec15 expression in predicting OS at one, two and three years. In order to visualize the data, the “ggplot2” package was utilized.

### Differentially expressed gene analysis

DEGs were identified among Siglec15 groups with different expression levels (high expression group: top 50%; low expression group: bottom 50%) in the TCGA database. Statistical analysis was performed using the “DEseq2” package. Up- and down-regulated DEGs were processed, adjusted for *P*-values < 0.05 and absolute log2 fold change (FC) > 1 for subsequent analysis and visualised using volcano and heat maps.

### Gene set enrichment analysis

Gene Ontology (GO) and Kyoto Encyclopedia of Genes and Genomes (KEGG) analyses were performed using R based on the identified DEGs. The “clusterProfiler” package was used for Gene set enrichment analysis (GSEA), which contains 1000 alignments and weighted enrichment statistics. Genes with a false discovery rate (FDR) < 0.25 and p.adjust < 0.05 were statistically significant and visualized using the “ggplot2” package.

### Glioma immune microenvironment analysis

Using the ‘ESTIMATE’ R package, we assessed the tumour purity, tumorpurity, immune score, and stromal score of glioma patients in the TCGA database. Based on the Cell type Identification by Estimating Relative Subsets of RNA Transcripts (CIBERSORT) algorithm, 22 types of immune cells were identified. For subsequent analysis, only samples with P < 0.05 in CIBERSORT were selected. The Wilcoxon rank-sum test was used to assess the significant differences in the proportion of immune infiltrating between the high and low expression of Siglec15. The levels of 29 immune-related and tumor-related markers were analysed by the “GSVA” R package using single sample gene set enrichment analysis (ssGSEA) in each sample. The association between the expression of Siglec15 and immune checkpoints and immune-related markers in TCGA glioma samples was also analysed.

### Immunofluorescence

FFPE tissue sections (4-µM in thickness) was deparaffinized, and rehydrated. Subsequently, antigen-retrieval was performed in the waterbath. Primary antibodies included Anti-Siglec15 (1:35; ab198684, Abcam) and Anti-CD163 (1:200; clone number [EPR19518]; ab182422, Abcam) were diluted in PBS containing 1% BSA. Slides were incubated overnight at 4°C, and then washed and incubated with corresponding secondary antibodies (1:1,000 of Goat anti-Mouse IgG (H+L) Cross-Adsorbed Secondary Antibody, Alexa Fluor 488 and Goat anti-Rabbit IgG (H+L) cross-Adsorbed secondary Antibody, Alexa Fluor 594) for 1 hour at room temperature and then washed. Slides were mounted with Fluorescent mounting medium (10105463, Dako, Glostrup, Denmark) and detected using a Zeiss fluorescence microscope (Image A2 Zeiss, Oberkochen, Germany).

### Statistical analysis

The statistical significance between two groups was tested using the Wilcoxon rank-sum test, and the Kruskal−Wallis test was used to compare multiple groups. Spearman’s correlation coefficient was used to analyse the correlation between Siglec15 expression and other immune-relevant genes. Student’s t test was used to determine the difference in double-tailed cell counts between disparate grades of gliomas. Based on a Kaplan–Meier analysis and a two-sided log-rank test, we determined the prognostic significance of variables univariately. In order to evaluate the effect of multiple independent prognostic factors on survival outcome, a Cox proportional-hazards model was applied in a stepwise manner. All statistical analyses were performed using R software (version 4.1.3/3.6.3), and two-tailed P < 0.05 was considered statistically significant.

## Results

### The upregulation of Siglec15 expression is associated with the malignant phenotype of glioma

**Figure 1:**
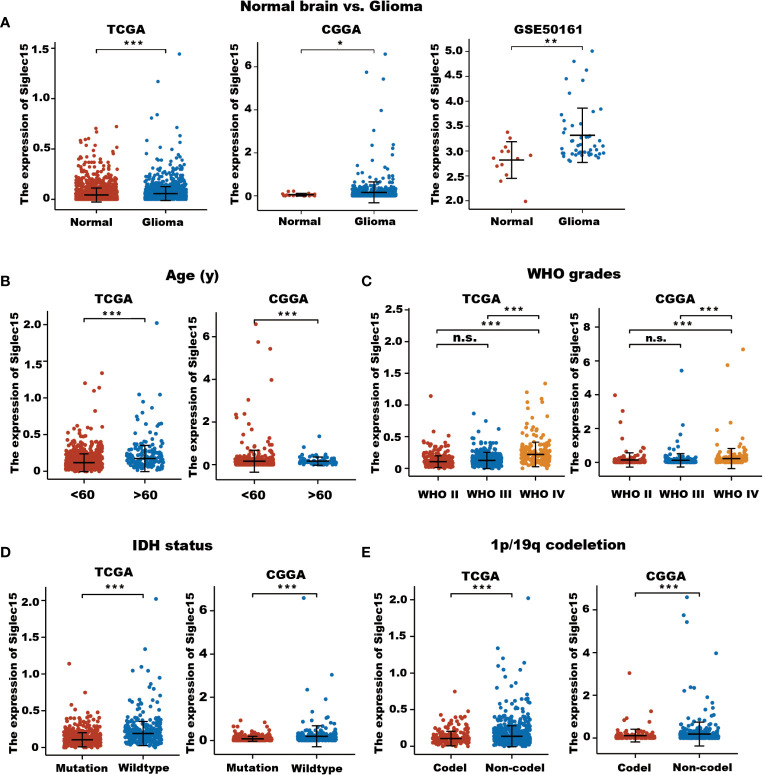
Expression level of Siglec15 mRNA in glioma and normal brain tissues. **(A)** Siglec15 expression in TCGA, CGGA, and GEO gliomas and normal brain tissues with GTEx database as control. **(B)** Different Siglec15 mRNA expression levels between aged <60 years and aged >60 years groups in TCGA and CGGA glioma patients. **(C)** Siglec15 mRNA expression levels was higher in high-grade (WHO grade IV) than in low-grade (WHO grade II and III) gliomas. **(D)** Siglec15 mRNA was higher in gliomas with wildtype IDH than those with mutated IDH. **(E)** Siglec15 mRNA was higher in gliomas without 1p/19 codeletion than those with 1p/19q codeletion. TCGA, the cancer genome atlas; CGGA, Chinese gliomas genome atlas; IDH, isocitrate dehydrogenase. n.s. non-significant, *P <0.05, **P <0.01, ***P < 0.001.

First, Siglec15 mRNA expression levels were analysed in glioma tissues and normal brain tissues using data obtained from the TGGA, CGGA, and GEO databases. The charateristics of patients were shown in **Table 1**. It was found that Siglec15 transcript levels were elevated in gliomas compared with normal brain tissues (*P* < 0.05, **Figure 1A**). To explore the potential impact of Siglec15 in glioma progression, subgroups were analysed by stratifying patients with various clinical features in the CGGA and TCGA datasets.

In terms of age at diagnosis, we found significantly increased levels of Siglec15 in patients aged over 60 years (*P* < 0.001, **Figure 1B**). Although no significant difference in Siglec15 expression was revealed between grade II and grade III in the TGGA datasets, Siglec15 expression levels were significantly higher in GBM (grade IV) than in low-grade gliomas (grade II and III) (TCGA: WHO grade II vs III, P = 0.224; WHO grade II vs IV, P <0.001; WHO grade III vs IV, *P* < 0.001; CGGA: WHO grade II vs III, *P* = 1; WHO grade II vs IV, *P <*0.001; WHO grade III vs IV, *P* < 0.001, **Figure 1C**). In both the CGGA and TCGA databases, patients with higher levels of Siglec15 expression were related to wild-type IDH (*P <*0.001, **Figure 1D**). We then evaluated the potential association between Siglec15 expression and 1p/19q status in these two databases. Siglec15 was found to be upregulated in 1p/19q noncodeletion glioma tissues (*P* < 0.001, **Figure 1E**). Taken together, these findings demonstrated a positive correlation between high Siglec15 expression and the malignant phenotype, poor treatment efficacy, and worse clinical outcomes in gliomas.

### Increased Siglec15 expression correlated with shortened recurrence time and unfavorable prognosis of glioma patients

Since high Siglec15 expression may be predictive of the malignant phenotype of gliomas, we subsequently investigated the predictive value of Siglec15 expression in the clinical prognoses of glioma patients.

**Figure 2:**
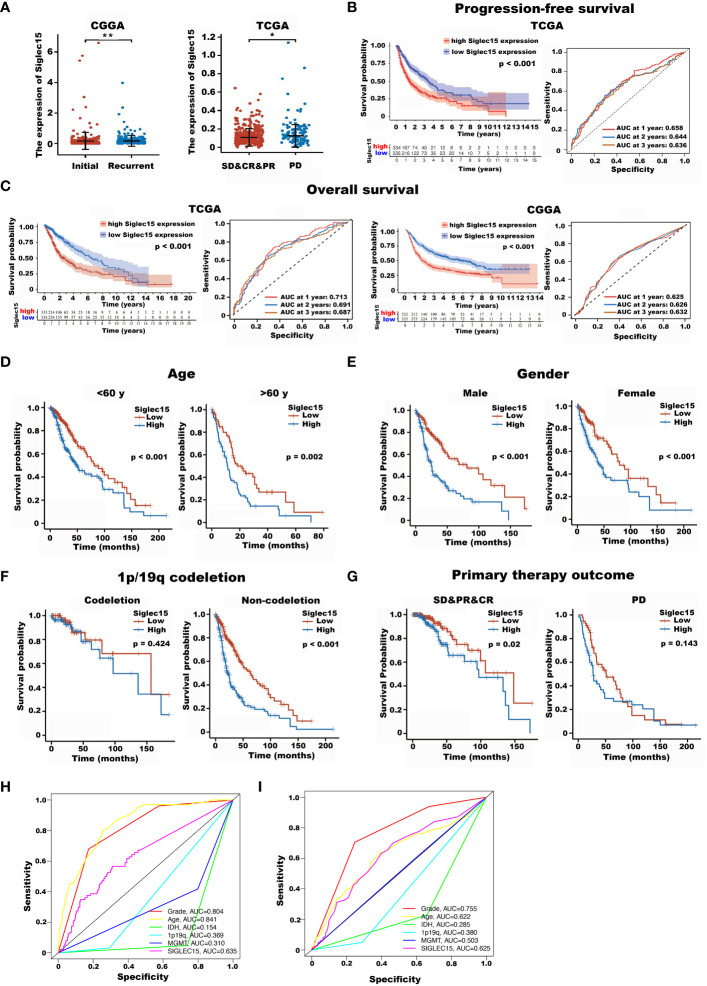
The impact of Siglec15 mRNA overexpression on the overall survival time and progression-free time of glioma patients. **(A)** Siglec15 mRNA expression levels were higher in recurrent than in initial glioma patients (left panel), and were higher in gliomas patients showed no response to treatment, compared with those were responded to treatment (right panel). **(B)** Glioma patients with high Siglec15 expression showed shortened progression-free time compared with those with low Siglec15 expression. **(C)** Glioma patients with high Siglec15 expression showed shortened overall survival time compared with those with low Siglec15 expression. **(D–G)** In different subgroups of gliomas patients stratified by various clinical characteristics including age **(D)**, gender **(E)**, 1p/19q codeletion status **(F)**, and response to initial treatment **(G)**, TCGA glioma patients with high Siglec15 expression all showed adverse overall survival time, except in 1p/19q codeletion and progressive disease subgroup. **(H)** Time-dependent ROCs for Siglec15 expression, grade, age, IDH, 1p19q and MGMT in glioma using TCGA database. **(I)** Time-dependent ROCs for Siglec15 expression, grade, age, IDH, 1p19q and MGMT in glioma using CGGA database. TCGA, the cancer genome atlas; CGGA, Chinese glioma genome atlas; SD, stable disease; PR, partial response; CR, complete response; PD, progressive disease; ROCs, receiver operation curves. *P <0.05, **P <0.01.

First, we compared Siglec15 expression between the initial and recurrent glioma tissues (**Figure 2A**) and between the patients with relatively malignant (progressive disease [PD]) and benign (stable disease [SD], complete response [CR] or partial response [PR]) clinical courses (**Figure 2A**). Siglec15 expression was found to be higher in recurrent gliomas as well as in those that did not respond to conventional resistance. These outcomes indicate that Siglec15 expression may be correlated with a more malignant phenotype and treatment resistance.

In terms of recurrence time, the results revealed that glioma patients with high Siglec15 expression had a shortened PFS compared with those with low expression (HR [95% CI], 1.87 [1.51–2.32], *P* < 0001, **Figure 2B**).

Next, we analysed the impact of the Siglec15 expression level on the OS of glioma patients. The results revealed that the OS of glioma patients with increased Siglec15 expression levels was unfavorable according to Kaplan−Meier survival analysis of TCGA database (*P* < 0.001) and CGGA database (*P* < 0.001) (**Figure 2C**). In addition, the ROC results showed that the AUC of Siglec15 expression in glioma patients was 0.728 for 1-year survival, 0.694 for 2-year survival and 0.686 for 3-year survival in the TCGA database and 0.623 for 1-year survival, 0.627 for 2-year survival and 0.632 for 3-year survival in the CGGA database. This evidence suggests that high levels of Siglec15 expression are associated with poor prognosis in patients with glioma.

To confirm the reliability of our findings on patient survival, we also examined the correlation between Siglec15 expression and OS in various subgroups of patients stratified by various clinical characteristics in the TCGA database.

The outcomes consistently revealed that patients with gliomas with the higher Siglec15 expression had significantly the poorer OS compared to those with a low Siglec15 level in subgroups including age, sex, 1p/19q status, and primary therapy outcome (**Figures 2D–G**). Interestingly, although there was no significant difference in the expression of Siglec15 between males and females, a high expression level of Siglec15 was remarkably associated with a poor OS in glioma patients in the male and female subgroups.

Furthermore, we performed time-dependent receiver operating characteristic (ROC) analysis. The area under the curve (AUC) of the ROC curve of Siglec15expression in glioma patients was 0.728 for 1-year survival, 0.694 for 2-year survival and 0.686 for 3-year survival in the TCGA database (**Figure 2H**) and 0.623 for 1-year survival, 0.627 for 2-year survival and 0.632 for 3-year survival in the CGGA database (**Figure 2I**). Collectively, high Siglec15 transcript levels indicate a worse clinical course and shortened recurrence. In addition, a clear positive result for age, grade and siglec15 can be seen in the multi-factorial ROC curve.

### Siglec15 protein expression was upregulated and was associated with adverse recurrence and survival time in gliomas

To validate the expression of Siglec15 and its impact on PFST and OST in gliomas, we subsequently performed immunohistochemistry in a series of glioma patients who received surgical resection in our hospital. Characteristics of the patients were summarized in **Table 2**.

**Table 2:** Characteristics of patients with glioma for immunohistochemical assess.

	Normal brain	Glioma			
		WHO II	WHO III	WHO IV	Total
Total no. of patients	6	30	25	37	92
Male	3	16	11	19	46
Female	3	14	14	18	46
Extent initial surgical resection (n)					
GTR		18	19	22	59
STR or biopsy		12	6	15	33
Radiotherapy					
Yes		23	18	30	71
No		7	7	7	21
Chemotherapy					
Yes		16	16	29	61
No		14	9	8	31
Siglec15 expression					
Low expression (TIS ≤ 4)	6	20	11	11	42
High expression (TIS > 4)	0	10	14	26	50
IDH-1/2 mutation					
Mutation		25	18	4	47
Wildtype		5	7	33	45
1p/19q co-deletion					
Co-deletion		1	3	5	9
Non-codeletion		1	6	15	22
N/A		28	16	17	61
TERT mutation					
Mutation		1	6	11	18
Wildtype		1	3	9	13
N/A		28	16	17	61
EGFR amplification					
Yes		0	2	9	10
No		2	7	11	21
N/A		28	16	17	61
CDKN2A/B homozygous loss					
Homozygous loss		0	1	4	5
Non-homozygous loss		2	8	16	26
N/A		28	16	17	61
MGMT promoter methylation					
Methylated		2	6	15	23
Unmethylated		0	3	5	8
N/A		28	16	17	61

Immunohistochemical analysis revealed that Siglec15 was not highly expressed in normal brain tissues but was overexpressed in 54.3% (50/92) of total gliomas. In more details, overexpression of Siglec15 was found in 33.3% (10/30) of WHO grade II, 56% (14/25) of WHO grade III, and 70.3% (26/37) of WHO grade IV gliomas, respectively (**Table 2**).

**Figure 3:**
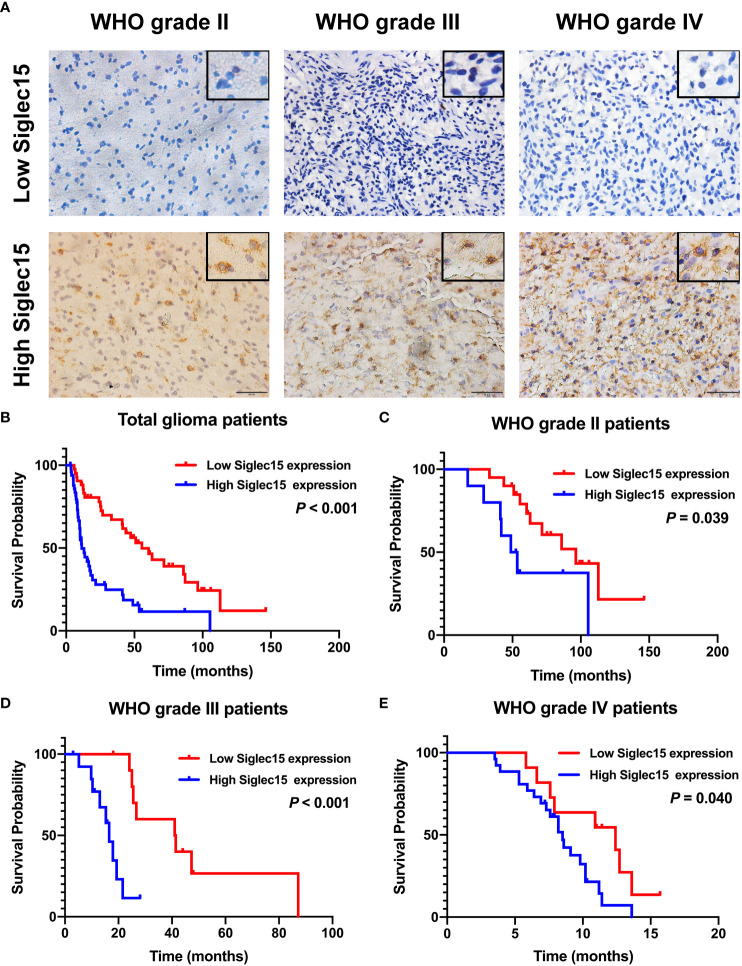
Representative images of immunohistochemical staining of Siglec15 in glioma and the impact of Siglec15 protein overexpression on the overall survival time of glioma patients. **(A)** Representative low and high Siglec15 expression in gliomas of different WHO grades by immunohistochemistry staining. **(B–E)** Siglec 15 overexpression was found to be associated with reduced overall survival time in total **(B)**, WHO grade II **(C)**, WHO grade III **(D)**, and WHO grade IV **(E)** glioma patients.

The expression of Siglec15 in various grades of glioma is shown in (**Figure 3A**). The log rank test showed that Siglec15 overexpression correlated with reduced OST in patients with gliomas(*P* < 0.001) (**Figure 3B**). Within subgroups divided by WHO grades, Siglec 15 overexpression was related with shortened OST in WHO grade II (*P* = 0.039) (**Figure 3C**), grade III (*P* < 0.001) (**Figure 3D**) and grade IV (*P* = 0.040) (**Figure 3E**), respectively. Moreover, the prognostic value of radiotherapy, chemotherapy, resection extent and malignancy was also revealed in total glioma patients (**Table 2**). Subsequently, we performed Cox multivariate analysis to stratify these variates and to validate prognostic value of Siglec15. Siglec15 protein overexpression was revealed to be an independent adverse prognostic indicator of OST [*P* = 0.039, hazard ratio 0.49, 95% CI (0.249–0.965)]. Additionally, radiotherapy (*P <*0.001), chemotherapy (*P* < 0.001), resection extent (*P* = 0.030), and malignancy (WHO grades, *P* < 0.001) were also found to serve as significantly independent indicators of OST (**Table 3**). Nevertheless, IDH-1 was found to serve as significantly independent indicator of OST only in univariate survival analysis (*P* < 0.01), but not in multivariate analysis (*P* = 0.852).

**Table 3:** Log rank test and Cox multivariate analysis.

	Log rank test		Cox multivariate analysis	
	Chi square	P*	HR (95% CI)	P*
Siglec15 overexpression	15.859	< 0.001*	0.48790 (0.24948–0.96556)	0.0369*
Resection extent	6644	0.010*	1.93127 (1.0651–3.502499)	0.031*
WHO grade	83.476	< 0.001*	51.863 (20.486–131.300)	< 0.001*
Radiotherapy	6.143	0.013*	3.606 (1.842–7.057)	< 0.001*
Chemotherapy	4.741	0.029*	11.728 (4.941–27.839)	< 0.001*
IDH1/2 mutation	21.171	<0.001*	1.073 (0.512–2.246)	0.852

*P < 0.05.

### Functional enrichment analysis of DEGs

**Figure 4:**
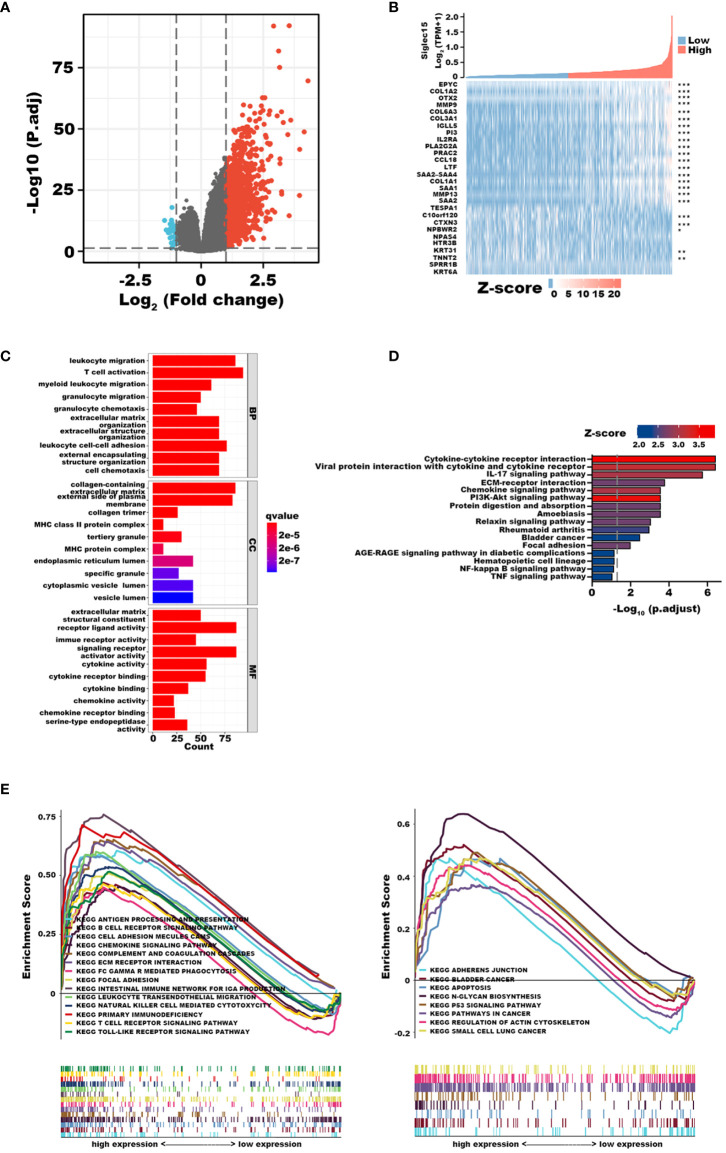
Functional Enrichment Analysis of differentially expressed genes (DEGs) derived from the cancer genome atlas (TCGA) database. **(A)** Volcano plot shows 1124 DEGs. **(B)** Heat maps showing top 18 upregulated and 10 downregulated DEGs. **(C)** Top 30 terms of Go enrichment analysis. **(D)** Top 16 terms of GSEA analysis. **(E)** Top 22 terms of KEGG enrichment analysis. *P < 0.05; **P < 0.01; ***P < 0.001.

To investigate the potential biological role of Siglec15 in gliomas, we identified DEGs between patients with high and low Siglec15 expressions. We identified a total of 1124 significantly different DEGs, of which 1093 were upregulated and 31 downregulated genes from the TCGA database (**Figures 4A, B**). GO enrichment analysis revealed that the DEGs were enriched in leukocyte migration, T-cell activation, MHC protein complex, immune receptor activity, cytokine activity, cytokine receptor binding, cytokine binding, chemokine activity, and chemokine receptor binding, among others (**Figure 4C**). In line with this, in KEGG analysis, we observed cytokine−cytokine receptor interactions, viral protein interactions with cytokines and cytokine receptors, the IL-17 signaling pathway, ECM-receptor interactions, the chemokine signaling pathway, the PI3K-Akt signaling pathway, protein digestion and absorption amoebiasis, the relaxin signaling pathway, rheumatoid arthritis, bladder cancer, focal adhesion, the AGE-RAGE signaling pathway in diabetic complications, hematopoietic cell lineage, the NF-kappa B signaling pathway and the TNF signaling pathway as underlying pathways in regulating Siglec15 expression (**Figure 4D**).

In addition, we completed GSEA to determine the possible biological functions of Siglec15 in gliomas. Correspondingly, enrichment analysis showed that upregulation of Siglec15 was related to leukocyte transendothelial migration, focal adhesion, ECM receptor interaction, and the T-cell receptor signaling pathway, in line with the results of GO and KEGG analyses (**Figure 4E**). Our findings highlighted the potential functions of Siglec15 in tumor immunity and ECM remodelling, allowing us to revisit its biological role in subsequent analyses.

### Siglec15 was related to immune cell infiltration

**Figure 5:**
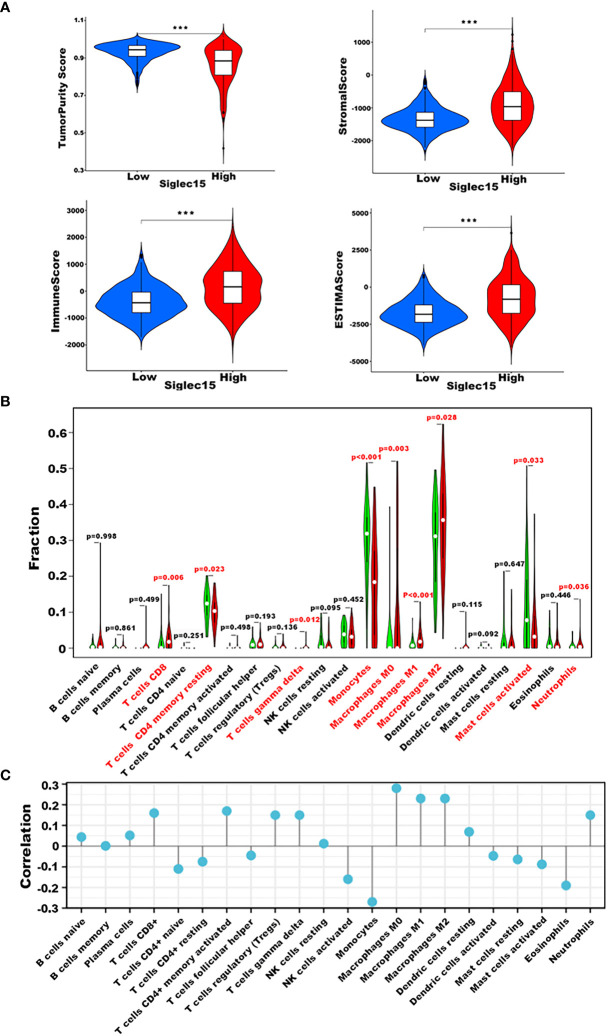
Siglec15-related Immune Cell Infiltration Analysis derived from TCGA database. **(A)** Correlation between Siglec15 expression with stromal score, immune score, tumorpurity, and ESTIMATE score. **(B)** Relationship between Siglec15 expression and presumed immune cell infiltration in glioma tissue. **(C)** Correlation analysis between Siglec15 expression level and CIBERSORT score of 22 immune cells. ***P < 0.001.

As mentioned previously, it was apparent that the elevated expression level of Siglec15 relates to adverse prognosis and immune response in glioma patients, and we subsequently elaborated on the effect of Siglec15 in reshaping the tumor microenvironment. First, we calculated the immune scores, tumorpurity, stromal scores and estimate scores, and these three scores were all positively correlated with Siglec15 expression (p <0.0001) among patients with glioma (**Figure 5A**). Next, we analysed the presumed immune cell infiltration in glioma tissue in TCGA datasets (**Figure 5B**). We found that CD8+ T cells, gamma delta T cells, M0 macrophages, M1 macrophages, M2 macrophages, and neutrophils infiltrated obviously more in glioma tissue, with Siglec15 being highly expressed, and resting memory CD4+ T cells, monocytes and activated mast cells remained markedly enriched in the low Siglec15 group. Additionally, we assessed the association between Siglec15 expression and immune cell infiltration levels to confirm our findings (**Figure 5C**). Consistently, the amount of infiltration of CD8+ T cells, activated memory CD4+ T cells, Tregs, gamma delta T cells, M0-, M1-, and M2 macrophages and neutrophils was positively correlated with the expression of Siglec15, while activated NK cells, monocytes and eosinophils were negatively correlated with the expression of Siglec15.

To validate these results, we counted 29 immune components in 698 glioma samples from TCGA using ssGSEA and compared the level of immune infiltration between the high and low Siglec15 expression groups. Further differential analysis of the level of immune infiltration revealed that the levels of immune infiltration of all 29 immune components were higher in the high Siglec15 expression subgroup than those in the low group (**Figure S2**).

### Association of Siglec15 with immunoregulatory, TAM-regulatory, and angiogenic genes

**Figure 6:**
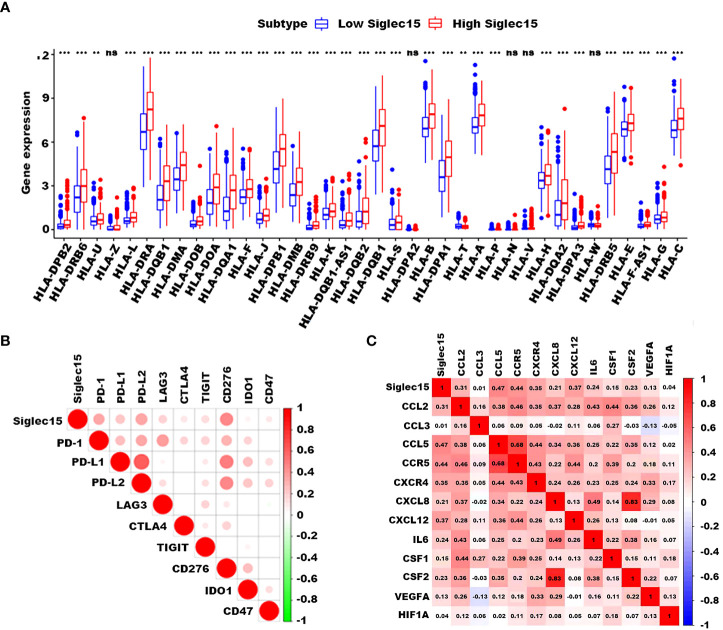
Correlation of Siglec15 expression with HLA-related, immunoregulatory, and angiogenic genes. **(A)** High Siglec15 expression correlated with a majority of HLA-related genes. **(B)** Siglec15 expression was closely related to a variety of immunoregulatory genes, including PD-1, PD-L1/2, LAG3, CTLA4, TIGIT, CD276, IDO1, and CD47. **(C)** Siglec15 correlated with genes which are critical in the regulation of recruitment, differentiation, and activation of TAMs and angiogenic genes. n.s. non-significant, **P<0.01, ***P < 0.001.

To investigate the role of Siglec15 in immune modulation, we examined the relationship between Siglec15 expression and immunoregulatory molecules in gliomas. We also discovered that the expression of a great majority of HLA-related genes was higher in the Siglec15 high expression group than that in the low expression group (**Figure 6A**). Correspondingly, Siglec15 could potentially interact with immune-related checkpoints, including PD1, PDL1, PDL2 and CD276 (B7-H3), implying a pivotal immunoregulatory role of Siglec15 in the glioma immune microenvironment (**Figure 6B**). In addition, Siglec15 was closely related to genes that are critical in the regulation of recruitment, differentiation, and activation of TAMs (CCL2, CCL3, CCL5, CCR5, IL-6, GM-CSF, VEFG, and CXCL8) and to angiogenic genes (SDF-1, CXCR4, HIF1a, CCL2, and VEGF), which are important targets in antiogenic therapy (**Figure 6C**).

### Increased Siglec15 expression on M2-like tumor-associated macrophages and N2-like tumor-associated neutrophils

**Figure 7:**
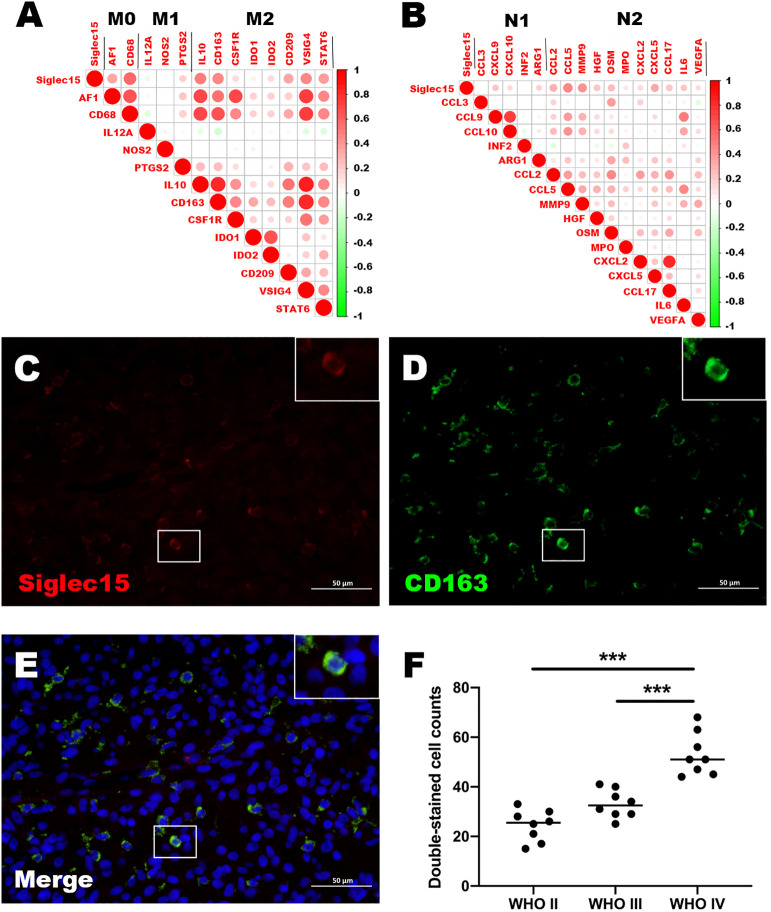
Relationship between Siglec15 expression and tumor-associated macrophages (TAMs) and tumor-associated neutrophils (TANs). **(A)** Siglec15 expression level was related to M2 TAMs. **(B)** Siglec15 was in association with N2 TANs. **(C–E)** Immunofluorescence shows that Siglec15 colocalize with CD163. **(F)** Double-stained cell counts (Siglec15 and CD163) in glioma with different grades. ***P < 0.001.

Based on CGGA and TCGA databases, we analyzed the correlation between expression of Siglec15 and phenotypic characteristics of macrophages and neutrophils. According to our findings, Siglec15 correlated extremely well with M0 and M2 markers of TAMs (M2-type macrophages promote tumor progression) rather than M1 markers (M1-type macrophages do not promote tumor progression) (**Figure 7A**). A similar correlation was found between Siglec15 and TANs’ N2 phenotype marker (**Figure 7B**). In the TME of gliomas, Siglec15 expression substantially alters immune cell infiltration, leading us to explore its cellular basis and distribution.

To confirm the expression of Silgec15 on M2 macrophages, the colocalization of Siglec15 and the well-established M2 macrophage marker CD163 was analysed using immunofluorescence. The results demonstrated that Siglec15 was substantially colocalized with CD163 (**Figures 7C–E**). In addition, the number of Siglec15+ CD163+ cells was higher in WHO grade IV gliomas than in grade II and III gliomas (**Figure 7F**).

Consequently, our results strongly indicated that Siglec15 expression was high in M2-like macrophages within the glioblastoma tumor microenvironment.

## Discussion

Despite the emergence of many novel treatment strategies based on standard surgical resection and chemoradiotherapy, such as targeted therapy and nanomaterial-based photodynamic and photothermal therapies, the overall survival of glioma patients has not improved significantly (12).

Glioma has an immunosuppressive nature, suppressing immunological surveillance against tumors. Checkpoint molecules, including PD-L1, CTLA-4, and IDO, have been demonstrated to be involved in the immune escape of glioma cells (13, 14). The introduction of checkpoint inhibitors has shown therapeutic efficacy by reducing tumor-infiltrating regulatory T (Treg) cell numbers and increasing overall survival (15). Despite the promising therapeutic effects of checkpoint inhibitors in preclinical studies of gliomas, the efficacy of these inhibitors is unsatisfactory in clinical settings (15). Therefore, deeper insight into the immunosuppressive microenvironment in glioma will aid in the improvement of immunotherapy.

Siglec15 belongs to the Siglec family, which includes important cell-surface transmembrane proteins with a characteristic sialic acid-binding immunoglobulin-type lectin structure (6). Siglec15 shows high structural homology with PD-L1, and the protein sequence of its extracellular domain bears 20%–30% identity to the B7 family (5). Siglec15 is crucial in the maintenance of immune homeostasis (5, 7). Additionally, Siglec15 acts as a critical immune suppressor with broad upregulation in a broad spectrum of malignancies (5). Currently, several Siglec15 inhibitors are undergoing clinical trials, including NextCure’s NC318 for solid cancer (phase II), Medimmune for AML (patent filed), and Daichi Sankyo’s DS-1501 (phase I) (16). Nonetheless, little is known about the expression and role of Siglec15 in gliomas. In our research, that Siglec15 is higher expressed in glioma than in normal tissues, which is the same as lung, break, head, and neck square cell carcinoma and bladder cancer (5, 17, 18). Furthermore, We report here in for the first time that Siglec15 is upregulated and associated with clinicopathological features such as ageing, higher WHO grade, IDH wildtype, 1p/19q non-coding, reduced PFST and OST, and infiltrating immunosuppressive cells in gliomas.

For decades, scholars worldwide have been in search of biomarkers of glioma that possess genetic predictors. Possessing predictors that influence prognosis provides good treatment strategies and helps clinical predictions for better clinical management and counselling (19). In this study, a high Siglec15 expression level was associated with an adverse prognosis and shortened recurrence, consistent with previous reports on many other tumors, including lung cancer (20), pancreatic ductal adenocarcinoma (21), breast cancer, thyroid carcinoma, sarcoma, and uterine corpus endometrial carcinoma (8). In addition, the impact of high Siglec15 expression levels on recurrence and overall survival was not influenced by age, sex, or 1p/19q codeletion. Further validation using immunohistochemistry confirmed that high Siglec15 expression could serve as a new biomarker and genetic predictor.

There is a growing body of research related to the role of gender and age on cancer incidence and survival. Although recent studies suggest that age alone does not predict survival in glioblastoma (22). However, higher age at diagnosis is the most powerful prognostic factor and is valid in all poor prognostic age groups, particularly in GBM (23). The prognosis for GBM worsens with increasing age (24). In cancer, there are significant gender differences in the incidence of most tumour types, suggesting that fundamental biological differences between males and females impact cancer incidence (25). The overall incidence rate and the incidence of most glioma subtypes were significantly higher in men compared to women in all age groups (26). Siglec15 has statistical significance in predicting adverse recurrence time and overall survival in different age and gender groups, which further illustrates the extensive application of Siglect15 as a prognostic indicator.

Together with stromal cells, immune cells, vascular endothelial cells, and their secreted factors and extracellular matrix (ECM) components, tumor cells can form a protumor progression microenvironment (27). Interestingly, we found that upregulated Siglec15 expression was associated with leukocyte transendothelial migration, focal adhesion, ECM receptor interaction, and T-cell receptor signaling pathways, indicating that Siglec15 might play a critical role in the immunosuppressive microenvironment of gliomas.

The ECM of tumor tissue is the noncellular component present within the tumor, providing physical support and activating the biochemical and biomechanical signals needed for tissue morphogenesis, differentiation and homeostasis (28). In GBM, the ECM is dramatically changed, and this altered ECM plays an important role in glioma cell invasion (29). In addition, the ECM controls many cellular activities through cytokines and chemokines, including morphogenesis, survival, differentiation, growth, migration, homeostasis, and immune function (28, 30).

Innate and adaptive immunity in the body are able to work in concert to identify and eliminate malignant cells. However, cancer cells can create various mechanisms to evade the immune system, thus allowing the tumor to progress to an advanced stage. Immune checkpoints are one of the mechanisms by which cancer cells camouflage themselves in the body. In our study, Siglec15 was broadly and positively correlated with immune checkpoints that have been reported as potential biomarkers of glioma, such as PD1, PDL1, PDL2, Lag3, CTLA4, TIGIT, CD276 (B7-H3), IDO1 and CD47. These results suggest a potential role and tandem effect of Siglec15 as a pivotal immune checkpoint in glioma. In addition, further evaluation using immune scores, matrix scores and evaluation scores also supported the relationship between Siglec15 expression and glioma immunity.

As the tumor progresses, immune cells infiltrating the tumor microenvironment not only exert antitumour effects but also promote immune evasion and tumor growth (31). Depending on the cytokine level in the tumor environment, macrophages can be classified as classically activated (M1, antitumour) or alternatively activated (M2, protumor) macrophages. Activated M2 macrophages contribute to immunosuppression, tumor growth and progression, and angiogenesis (32). Additionally, the number of TAMs in human tumors is associated with higher tumor grade and shorter survival in almost all tumors (33), except colon cancers (34). Currently, important precision molecular therapies in glioma include those targets involved in the regulation of recruitment, differentiation and activation of TAMs (CCL2, CCL3, CCL5, CCR5, IL-6, GM-CSF, CSF-1, VEGF and CXCL8); some of these molecules are already in the animal testing phase, such as CSF-1 (35). Interestingly, our results demonstrated that Siglec15 was broadly positively correlated with the expression of these genes, indirectly confirming its potential role in targeted macrophage therapy. Given the structural resemblance with the B7 family and dominant expression pattern on myeloid cells, it is reasonable to hypothesize that Siglec15 could exert an immunosuppressive effect through its expression on TAMs. In fact, a previous study on colon carcinoma validated the essential function of Siglec15 expressed by TAMs (16). Siglec15 blocking mAbs significantly inhibited tumor growth in mice inoculated with colon carcinoma cells mixed with wild-type bone marrow-derived macrophages (BMDMs) but not with Siglec15 knockout BMDMs (16).

T lymphocyte reactivation is an established therapeutic strategy for a variety of cancers (36). The focus on CD8 T cells as the central immune cell for tumor clearance is well reasoned. Nevertheless, CD8 T cell response may be not sufficient for an organ as immunologically unique as the brain. The anti-PD-1 therapy, however, did not show a helpful effect compared to standard therapy in a recent phase 3 clinical trial for patients with recurrent GBM (37). The diminished efficacy of anti-PD-1 therapy may be attributed to a number of factors, including infiltration of immunosuppressive myeloid cells, sequestration of T cells, release of inhibitory metabolites, and glucocorticoid-induced lymphopenia (38), which exhausts T cells (39). CD8 T cells in glioma do not play the same role as other tumors in clearing tumor cells for 2 main reasons: 1) CD8 cell possess the ability to directly kill tumor cells presenting tumor antigens *via* MHC I. However, there are a number of limitations to this approach in many cancers. Several cancers,including GBM, downregulate MHC I expression, as evidenced by the absence of MHC I expression on GBM cells that have invaded normal brain tissue (40); 2) There are multiple components of the glioma immune microenvironment, including myeloid-derived suppressor cells, that inhibit CD8T cell activation (41), TAM and other ingredients. TAMs are capable of expressing numerous compounds that act as antagonists to the isogenic receptors expressed on T cells, thereby reducing their ability to activate and proliferate. Additionally, TAMs release various inhibitory cytokines that impair T cell antitumour responses (42). Therefore, it is theoretically possible to use Siglec15 as an adjuvant therapy for PD-1 and CTLA-4 (43).

Despite their primary role in tissue homeostasis and host defense, neutrophils can also contribute to tumor formation in an altered state (44). It has been reported that TANs infiltrate human gliomas and that the degree of infiltration significantly correlates with tumor grade (45). N2 TANs promote tumor development and invasion and maintain cancer cell stem cells, angiogenesis and immunosuppression (46). It has been demonstrated in preclinical studies that N2 neutrophils support the expansion of the glioma stem cell pool by interacting with the S100 protein, thereby promoting glioblastoma progression (47). Thus, the correlation between Siglec15 and N2 TANs suggested that Siglec15 might also contribute to immunosuppression by overexpression on N2 TANs.

Previous studies have shown that macrophage/myeloid-associated Siglec-15 suppresses antigen-specific T-cell responses within the body (5).The function of Siglec15 affects both T cells and macrophages, which is consistent with our results. High Siglec15 expression is associated with M2 macrophage differentiation and may inhibit the CD4+ T-cell response in the immune microenvironment by being highly expressed on macrophages. Due to the immunosuppression experienced by glioma patients and the infiltration and polarization of macrophages and neutrophils that make glioma resistant to chemotherapy and radiation (48). It is reasonable to predict that Siglec15 is involved in glioma drug resistance through modulating the immune response.

Nevertheless, there are many limitations in our work. First, the sample size of the immunohistochemical and survival studies was not large enough. Second, immunohistochemistry might not accurately measure Siglec15 expression. Third, since the potential immunosuppressive role of Siglec15 in gliomas was obtained from bioinformatics analysis, efforts such as functional experiments need to be made to understand how Siglec15 dysregulation occurs in the tumor immune microenvironment and to determine whether Siglec15 inhibitors may have therapeutic effects in gliomas in preclinical studies. This might be helpful to develop a novel and effective immunotherapy strategy and benefit glioma patients with improved prognoses.

## Conclusion

We find that Siglec15 overexpression is an unfavorable prognostic biomarker and potentially plays a significant role in the tumor microenvironment of gliomas. High Siglec15 expression is associated with M2 macrophage polarzation and may inhibit the CD4+ T-cell response in the immune microenvironment by being highly expressed on macrophages. Apart from this, the expression of Siglec15 on glioma cells and immune cells may mediate immune escape through cell adhesion. Thus, Siglec15 may serve as a potentially pivotal immune checkpoint for glioma. In conclusion, our research provides a theoretical basis for the possibility of Siglec15 inhibitors as a synergistic or alternative therapeutic option to the existing immune checkpoint inhibitors.

## Data availability statement

The original contributions presented in the study are included in the article/**Supplementary Material**. Further inquiries can be directed to the corresponding authors.

## Ethics statement

The studies involving human participants were reviewed and approved by Research Ethics Committee of Shandong Provincial Hospital Affiliated to Shandong First Medical University. The patients/participants provided their written informed consent to participate in this study.

## Author contributions

JW and BL conceived study design. SX, LX, QD, and KW collected data. JW, SX, LX, BL, and QD participated in data analysis. JW, and LX drafted the paper. BL, and SX participated in revision of the paper. All authors contributed to the article and approved the submitted version.
