# Supplementary material for: Siglec15 is a prognostic indicator and a potential tumor-related macrophage regulator that is involved in the suppressive immunomicroenvironment in gliomas
Source: Front Immunol. 2023 May 30;14:1065062. doi: 10.3389/fimmu.2023.1065062 (PMC10266207; doi:10.3389/fimmu.2023.1065062)
Supplement: Supplementary file 1 [file DataSheet_1.docx]

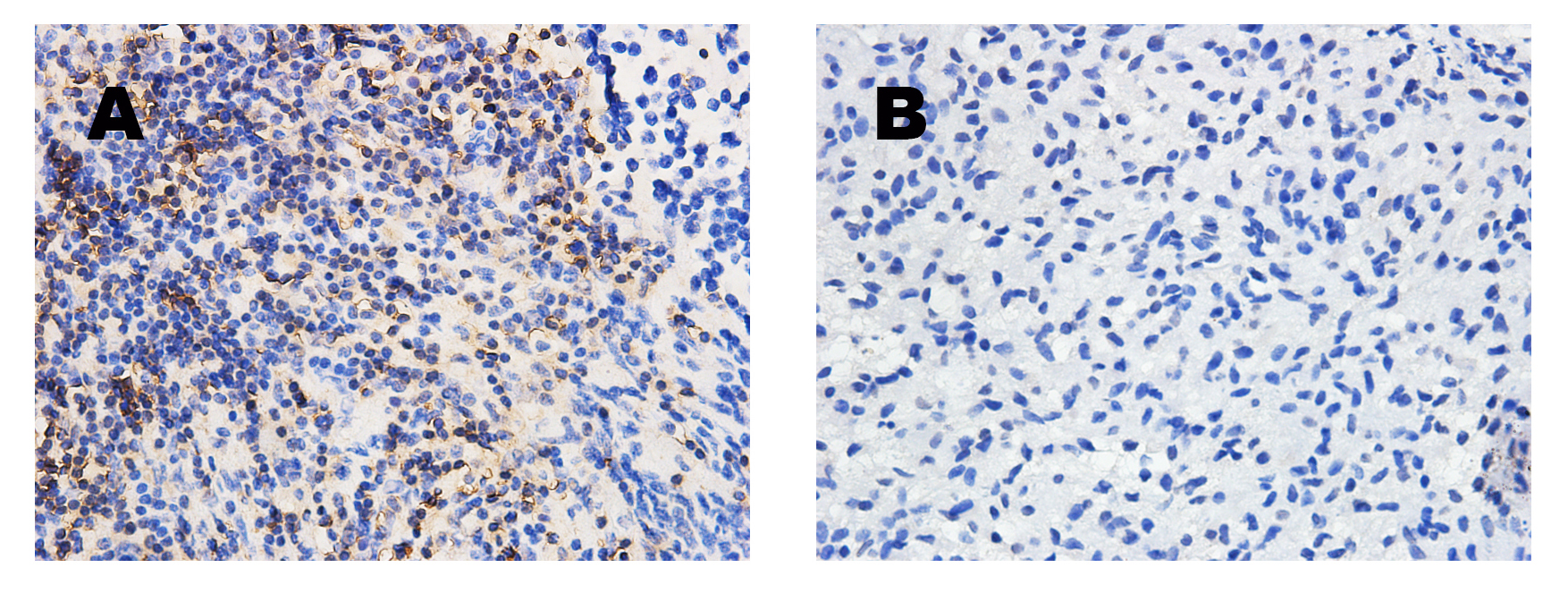


**Figure S1** Representative images of positive and negative control for Siglec15 immunostaining in immunohistochemistry. A: Human medulloblastoma tissues serves as positive control. B: Slides were incubated with phosphate buffered saline in place of the primary antibodies to serve as a negative control.


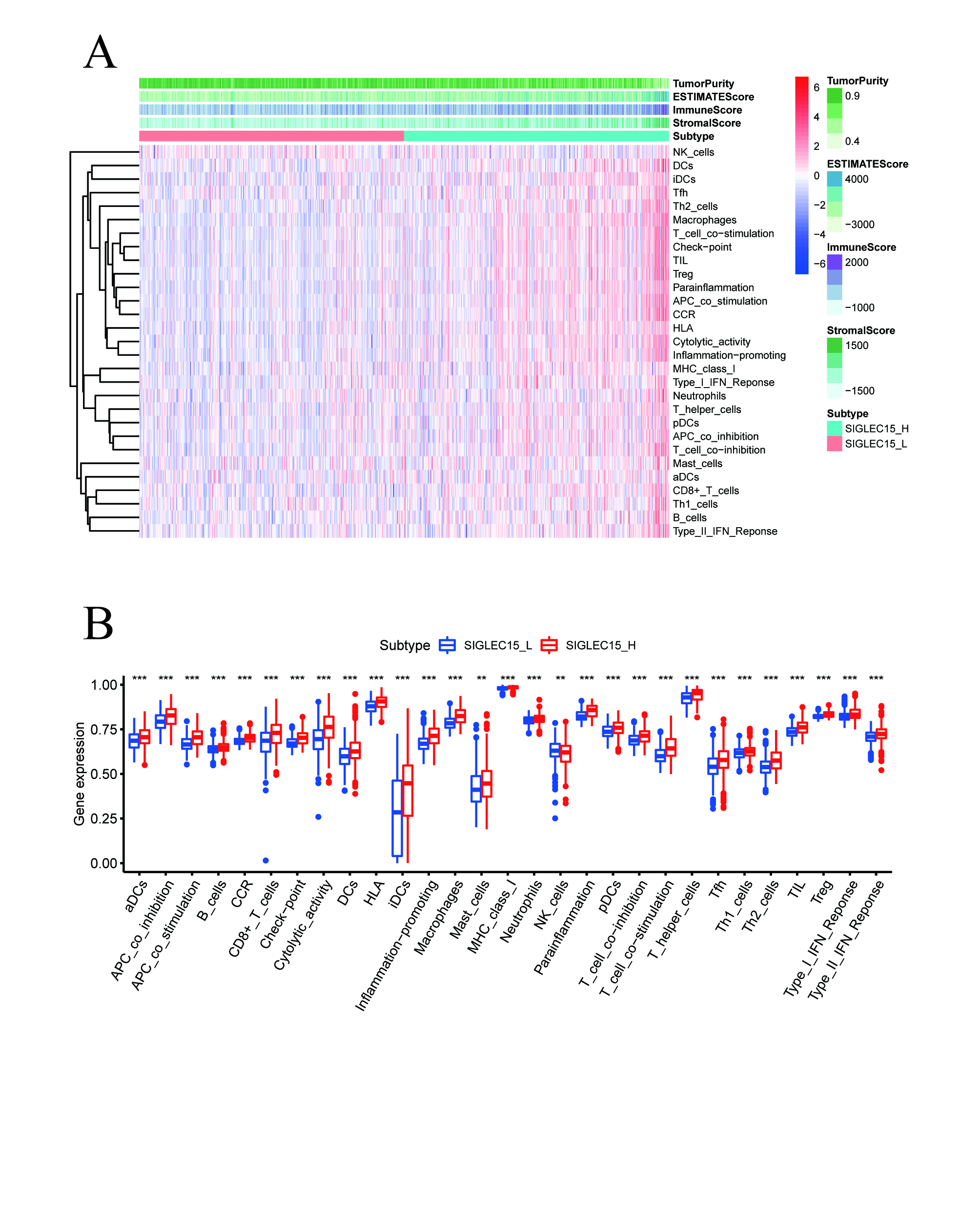
**Figure S2**  High Siglec15 expression is associated with glioma immune cell infiltration and immune components.
